# *De Novo* Transcriptome Assembly for the Tropical Grass *Panicum maximum* Jacq

**DOI:** 10.1371/journal.pone.0070781

**Published:** 2013-07-29

**Authors:** Guilherme Toledo-Silva, Claudio Benicio Cardoso-Silva, Liana Jank, Anete Pereira Souza

**Affiliations:** 1 Molecular Biology Center and Genetic Engineering (CBMEG), University of Campinas (UNICAMP), Campinas, São Paulo, Brazil; 2 Embrapa Beef Cattle, Campo Grande, Mato Grosso do Sul, Brazil; 3 Department of Plant Biology, Biology Institute, University of Campinas (UNICAMP), Campinas, São Paulo, Brazil; Auburn University, United States of America

## Abstract

Guinea grass (*Panicum maximum* Jacq.) is a tropical African grass often used to feed beef cattle, which is an important economic activity in Brazil. Brazil is the leader in global meat exportation because of its exclusively pasture-raised bovine herds. Guinea grass also has potential uses in bioenergy production due to its elevated biomass generation through the C_4_ photosynthesis pathway. We generated approximately 13 Gb of data from Illumina sequencing of *P. maximum* leaves. Four different genotypes were sequenced, and the combined reads were assembled *de novo* into 38,192 unigenes and annotated; approximately 63% of the unigenes had homology to other proteins in the NCBI non-redundant protein database. Functional classification through COG (Clusters of Orthologous Groups), GO (Gene Ontology) and KEGG (Kyoto Encyclopedia of Genes and Genomes) analyses showed that the unigenes from Guinea grass leaves are involved in a wide range of biological processes and metabolic pathways, including C_4_ photosynthesis and lignocellulose generation, which are important for cattle grazing and bioenergy production. The most abundant transcripts were involved in carbon fixation, photosynthesis, RNA translation and heavy metal cellular homeostasis. Finally, we identified a number of potential molecular markers, including 5,035 microsatellites (SSRs) and 346,456 single nucleotide polymorphisms (SNPs). To the best of our knowledge, this is the first study to characterize the complete leaf transcriptome of *P. maximum* using high-throughput sequencing. The biological information provided here will aid in gene expression studies and marker-assisted selection-based breeding research in tropical grasses.

## Background

Approximately half of the world’s bovine meat is produced in tropical or subtropical areas [1]. Pastures serve as the basis for beef production in Brazil and occupy an area of 101.4 million hectares [2], which, when considered together with natural pastures, is similar to the area occupied by crops and forests [3]. Additionally, Brazilian commercial beef cattle herds are the largest in the world, and Brazil is the leader in global bovine meat exports [1]. Bovine meat production in Brazil is performed exclusively on pasture, according to international market concerns regarding food security [3]. The primary grasses used as forage for cattle belong to species of *Brachiaria* or *Panicum maximum* [4]. Guinea grass is a forage grass that is native to Eastern and Southern Africa and is found in tropical and subtropical regions [5]. African forage grasses evolved in the presence of large mammals, so they are able to tolerate intense grazing and are very productive, vigorous and robust [3]. *Panicum maximum* is important for pasture and for green silage and hay production in the tropical Americas because of its high yield and nutritional content. Guinea grass belongs to the family Poaceae, subfamily Panicoideae and tribe Paniceae and constitutes an agamic complex with *P. infestum* Anders and *P. trichocladum* K. Schum [5]. *Panicum maximum* is adaptable to diverse ecosystems and is grown in several countries [6]. It is the most productive seed-propagated tropical forage grass and the second most cultivated forage grass in Brazil [7]. However, most of the land is cultivated by just a few clonal genotypes, which presents a considerable risk for livestock pasture-based systems. The development of new forage grass cultivars with adaptability to the various edaphic and climatic conditions found in Brazil to enhance the diversity of forage grasses is a top priority of breeding programs [3]. *Panicum maximum* is a tetraploid species (2n = 4× = 32) of autopolyploid origin [8] that undergoes gametophytic aposporous apomictic reproduction [9]. Plants produced by apomixis are genetically identical to the mother plant as a result of clonal propagation by seeds [3]. However, a few genotypes found in natural populations exhibit diploidy (2n = 2× = 16) and a sexual reproductive mode [6]. Sexual *P. maximum* are important in breeding programs because they represent an effective tool for breeding and promoting diversification. Tetraploidy can be artificially induced in diploid plants to permit hybridization with apomictic tetraploid genotypes. Apomixis in *P. maximum* is determined by a gene or group of genes through simple inheritance, and the progeny of a cross between sexual and apomictic plants will exhibit a 1∶1 ratio of sexual and apomictic plants [6]. The inheritance of apomixis allows the fixation of desirable F_1_ hybrids through cloning to generate heterosis and advantageous heterogeneous gene combinations, with subsequent seed production and commercialization [9].

Tropical forage grasses exhibit high growth rates and biomass yields as a consequence of the C_4_ photosynthetic pathway [4]. C_4_ photosynthesis involves several biochemical and anatomical adjustments to accumulate additional CO_2_ compared with C_3_ photosynthesis, using the key enzyme Rubisco. Grasses comprise the majority of C_4_ plants (4,500 species), followed by sedges (1,500 species) and dicots (1,200 species). The importance of C_4_ plants arises mainly from their global primary productivity; they account for approximately a quarter of total production [10]. In addition to their benefits for livestock, grasses such as *P. maximum* have potential as alternative energy sources through energy production via biomass generation, and this use is also considered in the species breeding objectives [3]. Species such as *Panicum virgatum* are being intensely studied for energy production [11]–[13]. Currently, the breeding of tropical forage grasses such as *P. maximum* and several *Brachiaria* species is based primarily on the assessment and selection of natural genotypes represented in germplasm banks, using the variability obtained from grasses of African origin [14], [15]. The development of more productive and efficient cultivars can be improved through the use of genomics, transcriptomics and proteomics. The characterization of molecular markers is important for marker-assisted selection, germplasm assessment, the identification of hybrids and genome mapping [3]. Studies focusing on the molecular biology of *P. maximum*, including the characterization of molecular markers, genetic profiling, the search for apomixis-related genes and genetic evaluation of germplasm collections, have been performed [7], [15]–[20]. However, knowledge about the Guinea grass transcriptome remains limited, and very few *P. maximum* protein and nucleotide sequences are available in current databases. The identification of transcribed regions of the genome using high-throughput sequencing (RNA-seq) provides a viable alternative for the analysis of non-model organisms with large genome sizes. RNA-seq methodology allows researchers to study the transcriptomes of non-model species and assess gene expression and patterns of regulation [21]. RNA-seq also allows the discovery of putative molecular markers, such as microsatellites (SSRs) and single nucleotide polymorphisms (SNPs), because of the large quantity of data produced. Here, we present an overview of the transcriptome of *P. maximum* leaves. We constructed libraries from each of four genotypes currently used in the Guinea grass breeding program. We matched these sequences to known proteins in several databases using BLAST searches. Protein matches included a number of genes relevant to C_4_ photosynthesis and lignocellulose biosynthesis. Furthermore, we screened the transcriptome for putative SSRs and SNPs, which will allow genome-wide screening of variation among different genotypes. The resulting assembled and annotated transcriptome sequences constitute a comprehensive genomic resource available for further studies and may enable the rapid identification of genes that are involved in pathways important for beef cattle grazing and potential biomass energy production.

## Materials and Methods

### Plant Material and RNA Extraction

Four different *P. maximum* genotypes were sampled from the Embrapa Beef Cattle collection (Campo Grande, MS, Brazil). The institution maintains a collection of Guinea grass germplasm and performs breeding research. The plants chosen for this study were the two sexual accessions that appeared the most promising after germplasm evaluation and two apomictic accessions that have been released as commercial cultivars [3]. The S10 and S12 genotypes were originally diploid with sexual reproduction and were tetraploidized using colchicine for crossing with tetraploid apomictic accessions. We used the tetraploid genotypes S10 and S12 for this study. The Tanzania and Mombaça genotypes are commercial cultivars of *P. maximum* that are autotetraploid with apomictic reproduction and desirable agronomic characteristics. Total RNA was isolated from the leaves using a modified lithium chloride protocol [22]. RNA quality was measured on a 2100 Bioanalyzer (Agilent Technologies, Palo Alto, CA).

### RNA-Seq Library Preparation and Sequencing

A transcript library was constructed using a TruSeq RNA sample preparation kit (Illumina Inc., San Diego, CA) according to the manufacturer’s instructions. The quality of the library was assessed on a 2100 Bioanalyzer (Agilent Technologies, Palo Alto, CA) and clustered using a TruSeq PE Cluster Kit on cBot (Illumina Inc., San Diego, CA). The samples were sequenced on an Illumina GAIIx using TruSeq SBS 36-Cycle kits (Illumina, San Diego, CA).

### Raw Data Analysis and De Novo Transcriptome Assembly

High-quality reads (minimum 30 phred score) were filtered using a NGS QC Toolkit 2.3 [23]. Only paired-end reads (PE) from the four sequenced samples were assembled using Trinity software [24], which used three independent modules (Inchworm, Butterfly and Chrysalis) to assemble the transcriptome sequencing data *de novo*. Data from the four genotypes were assembled together to obtain a reference transcriptome of *P. maximum* leaves. Short transcripts (<300 bp) were discarded from the resulting assembly. The Bowtie aligner [25] with default parameters was used to map the reads back onto transcripts. Our criteria for determining non-redundant contigs (unigenes) for transcriptome annotation considered the first Butterfly transcript generated per Chrysalis component as representative. Partial and complete open reading frames (ORFs) were predicted using the transdecoder script present in the Trinity package, with a minimum length of 200 amino acids. All reads were deposited in the NCBI Short Read Archive (SRA) under accession number SRR821833.

### Annotation

Unigenes were used as queries to search protein databases using the BLAST+ program [26]. The queried databases included the NCBI non-redundant protein database (nr), UniProtKB-SwissProt, Clusters of Orthologous Groups (COG) and Phytozome grass data (www.phytozome.net); the Phytozome database includes protein sequences from *Sorghum bicolor*, *Zea mays*, *Setaria italica*, *Panicum virgatum*, *Oryza sativa* and *Brachypodium distachyon*. Homology searches applied BLASTx with an e-value cut-off of 1E-06 (in the case of the COG database, 1E-20 was used for increased stringency). Gene ontology (GO) terms were obtained from nr hits using Blast2GO software [27] with default parameters for the mapping and annotation steps, except that an e-value cutoff of 1E-10 was used for BLAST hits in the annotation step. Go-slim with plant slim (*Arabidopsis thaliana*) as an alias was used to summarize the GO term annotations of the transcriptome. WEGO [28] was used to functionally classify GO terms and graphically represent the distribution of unigene functions. The metabolic pathways were mapped using the Kyoto Encyclopedia of Genes and Genomes (KEGG) Automatic Annotation Server (KAAS) [29] with a bi-directional best-hit strategy to assign KEGG orthology terms (KO) to unigenes. The identified pathways were settled using their respective KO assignments.

### Abundance Estimation

RSEM (RNA-Seq by Expectation Maximization) software [30] was used to estimate the unigene FPKM values (fragments per kilobase of transcript per million mapped reads) based on read abundance using the Bowtie [25] aligner. Our discrimination of unique and shared transcripts among the sequenced plants was based on their FPKM values; only transcripts with FPKM values higher than 0.5 were considered.

### Variant Detection

Microsatellites were searched using the MISA script [31], with the motif rules set to a six-copy minimum for dinucleotides and a four-copy minimum for trinucleotides to hexanucleotides, guaranteeing a minimum length of 12 bp. As shown in yeast [32], SSRs with lengths of less than 12 bp exhibit non-deterministic variation and have a mutation potential similar to that of non-microsatellite regions. Burrows-Wheeler Aligner (BWA) [33] was used to align the reads back to the transcripts. Sequences from individual plants were mapped against unigenes. The default parameters were used to map the PE reads. The resulting alignment was analyzed using the Base Alignment Quality (BAQ) function of SAMtools [34], which provides an efficient and effective method for ruling out false SNPs caused by nearby insertions and/or deletions (INDELs). Subsequently, we used Freebayes [35] for variant calling using the following parameters: ploidy 4, minimum read counting for variant calling 2, minimum base quality 30, minimum mapping quality 20, minimum coverage 20 and no INDELs or multiple nucleotide polymorphisms (MNPs) called. Variants from sequenced genotypes were compared using the vcf-isec program from VCFtools [36], which generated a list of unique and shared SNPs.

### Ethics Statement

We certify that no specific permits were required for the described field studies. This work was a collaborative research project developed by researchers from UNICAMP (Brazil) and EMBRAPA Beef Cattle (Brazil). Additionally, we confirm that the field studies did not involve endangered or protected species.

## Results and Discussion

### Sequencing and Assembling

**Figure 1:**
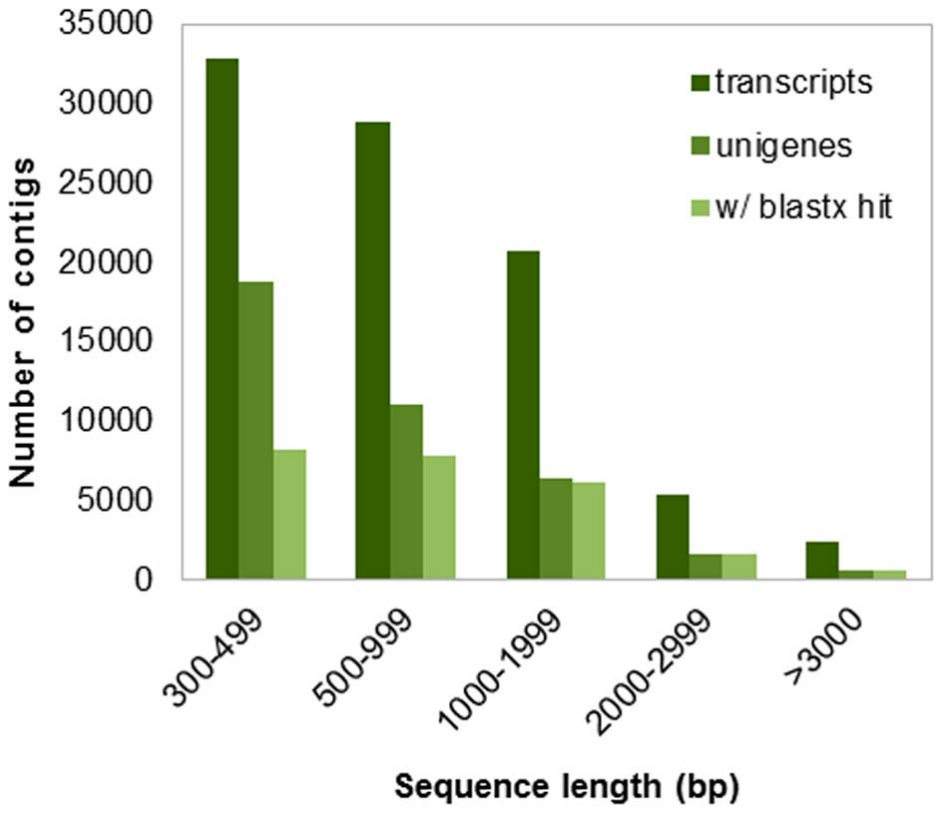
*De novo* assembly length distribution. Histogram of the sequence-length distribution of transcripts, unigenes and unigenes with significant BLASTx hits in the NCBI nr database.

We produced a total of 168,053,718 PE reads using Illumina technology, which generated 13.44 Gb of data (Table 1). No significant differences in sequencing data characteristics were observed among individuals (Table S1). After quality assessment and data filtering, 120,838,336 reads (96.32% Q20 bases and 47.32% GC content) were selected for *de novo* assembly. Using Trinity software [24], 88,292 transcripts were assembled, with a mean length of 942 bp and an N50 length of 1,272 bp. The Bowtie aligner [25] mapped 85.96% of the reads onto assembled sequences, considering only properly mapped paired-ends. We selected 38,192 sequences (43.25% of total transcripts) as unigenes, with a mean length of 758 bp and an N50 of 981 bp. The length distribution of the transcripts and unigenes is shown in Figure 1. We found that the average GC content of the *P. maximum* unigenes was 48.42%, slightly higher than the mean GC content of the raw reads. The average length of the unigenes was similar to that of chili pepper (712 bp) [37] and Ma bamboo (735 bp) unigenes [38] and higher than those of switchgrass (535 bp) [39] and safflower (446 bp) unigenes [40]. The N50 value of the *P. maximum* unigenes was shorter than those of chili pepper (1,076 bp) and Ma bamboo (1,132 bp) and higher than that of safflower (555 bp). The *P. maximum* GC content was similar to that of rice (47.52%) and slightly higher than that of *A. thaliana* (41.10%) [41]. Direct comparisons of assembly metrics were challenging because the methods used for unigene definition and/or minimum contig settings have not been standardized. Nevertheless, these metrics showed that the current assembly was successful in obtaining useful leaf transcripts of *P. maximum*. The transdecoder script from the Trinity package was used to predict ORFs in the transcripts and unigenes. The total ORFs represented 31.04% of the assembled transcripts, whereas this value was slightly lower for unigenes (29.32%). A summary of the sequencing, assembly and ORF prediction process is presented in Table 1.

**Table 1:** Summary of assembled transcripts and unigenes of *P. maximum* leaves.

Total raw reads	168,053,718
Total data	13.44 Gb
Total clean reads	120,838,336
Q20 bases	96.32%
GC percentage	47.32%
Total transcripts	88.292
Transcripts mean length	942 bp
Transcripts N50	1272 bp
PE mapped reads (Bowtie)	85.96%
Unigenes	38.192
Unigenes mean length	758 bp
Unigenes N50	981 bp
Unigenes GC percentage	48.42%
Predicted ORFs (transcripts)	31.04%
Predicted ORFs (unigenes)	29.32%

### Annotation

**Figure 2:**
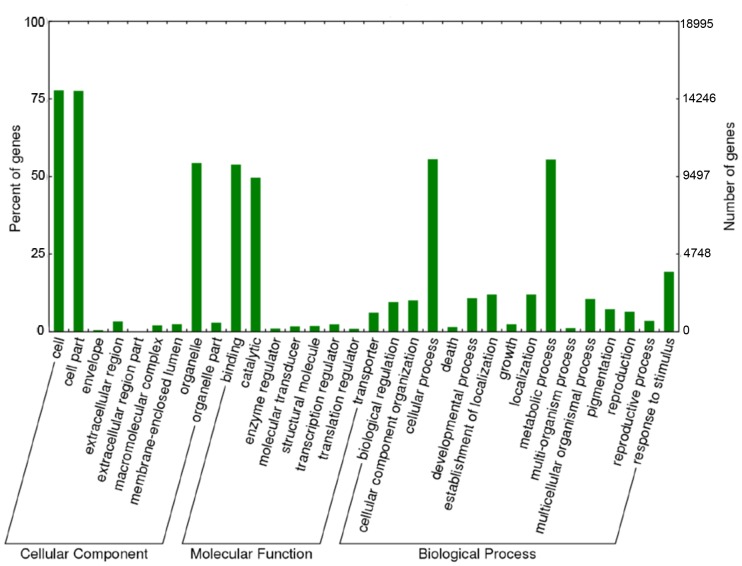
Gene ontology classification of *Panicum maximum* unigenes. Distribution of the GO categories assigned to the *P. maximum* transcriptome. Transcripts were classified into three categories: cellular components, molecular functions and biological processes.

The 38,192 assembled unigenes were queried against different protein databases, as shown in Table 2. We found 24,122 sequences (63.15%) similar to proteins in the nr database. The top-hit species distribution is presented in Figure S1. Among the nr BLASTx top hits, 10,851 were *Sorghum bicolor* proteins, followed by *Zea mays* (7,581), *Oryza sativa* (3,372), *Brachypodium distachyon* (1,020) and *Hordeum vulgare* (509). These five species accounted for ∼96% of the total nr top hits, which was expected because these species are closely related to *P. maximum*. Additionally, as shown in Figure 1, there was a strong correlation between transcript length and annotation success; 53.59% of the sequences between 300–999 bp in length were successfully annotated, whereas 96.79% of the longer transcripts (>1,000 bp) retrieved hits above the e-value cutoff. A search for homology against the manually curated UniProtKB-SwissProt database produced 16,396 hits (42.93%) because of the smaller number of proteins in this more reliable protein bank. The Phytozome grass protein database comparison displayed a slightly higher number of hits (26,319, 68.91%) than the nr search, mostly because it contained *P. virgatum* and *S. italica* protein sequences that were not present in the nr database. The top hit species in searches of the grass database were *S. italica* (44.40%), *P. virgatum* (31.87%) and *S. bicolor* (10.11%). A homology search against the COG protein database returned 22,473 hits (58.84%). The COG database was used to define the orthologous functions of unigenes, as shown in Figure S2. The functional classification of the COG classes was inferred from a BLAST homology search against the COG protein database. COG ortholog classes were determined by comparing the protein sequences of complete genomes representing major phylogenetic lineages. Each COG class consisted of individual proteins or groups of paralogs from at least three lineages and thus corresponded to an ancient conserved domain [42]. Unigenes were classified into 25 functional categories and 8,850 COG terms. The top categories among the COG terms were general function prediction only (1,515) and DNA replication, recombination and repair (1,484). Within the general COG classification (Figure S3), unigenes with matched COG terms were distributed into information storage and processing (2,575; 29%), cellular processes (1,697; 19%), metabolism (2,503; 28%) and poorly characterized (2,075; 24%). The COG category classifications showed that the identified leaf transcripts were involved in a wide range of functions and used in a great number of processes that configure the tissue metabolism transcriptional machinery. The results of BLASTx searches against the nr protein database were imported into Blast2GO [27] for GO mapping and annotation. Based on the nr 20 top hits, the Blast2GO program obtained GO annotations for the unigenes, and WEGO software [28] was used to perform GO functional classification into the three major classes. Among the unigenes with nr hits, 18,995 (49.73%) were assigned to gene ontology classes with 100,440 functional terms. Biological processes comprised the majority of the functional terms (44,880; 45%), followed by cellular components (28,647; 28%) and molecular functions (26,913; 27%) (Figures 2 and S3). The distribution of GO functional classifications among unigenes was similar to the distribution in *P. virgatum* [43]. Within the biological processes category, cellular (10,556 unigenes) and metabolic (10,542 unigenes) processes were prominently represented. Additionally, response to stimulus (3,664 unigenes) was an abundant biological process term, and response to stress (2,658 unigenes) was the most highly represented child GO term. Of particular interest, 1,467 unigenes were annotated as related to reproductive processes. These unigenes may provide valuable information for further studies of reproduction in *P. maximum*. These sequences provide new information about the genes involved in this process, which will aid future studies focusing on understanding Guinea grass reproduction and transferring *Panicum*-type apomixis and/or expressing this phenotype in grain crops. This type of apomixis can potentially be expressed in grain crops because it maintains the female to male genome ratio required for seed endosperm development [9]. In summary, 195 different terms were found among the unigenes, indicating that the genes expressed in Guinea grass leaves are involved in a wide variety of biological functions, as indicated by their COG classification. In the cellular component category, 230 terms were successfully mapped, with cell (14,765) and cell part (14,732) comprising the majority of this category. Among the molecular function categories, binding (10,235) and catalytic activity (9,435) were the most represented among the 33 terms found.

**Table 2:** Annotation summary of 38,192 *P. maximum* unigenes.

Database	Hits	Hits percentage
NCBI non-redundant proteins (nr)	24,122	63.15%
SwissProt	16,396	42.93%
COG	22,473	58.84%
KEGG	4,110	10.76%
Grass	26,319	68.92%
Gene ontology	18,995	49.73%

To correlate *P. maximum* leaf unigenes with known metabolic pathways, we used the KAAS server to assign sequences with KEGG orthology (KO) terms and their respective KEGG maps. A total of 4,110 (10.76%) assembled unigenes were associated with 2,665 KO terms and 297 pathways. Highly represented pathways included metabolic pathways (681 members) and secondary metabolite biosynthesis pathways (313 members). Similarly, pathways with the potential for further studies were detected using KAAS annotation; these pathways included glycolysis/gluconeogenesis (31 members) (Figure S4), photosynthesis (28 members) (Figure S5), carbon fixation in photosynthetic organisms (23 members) (Figure S6), phenylpropanoid biosynthesis (15 members) (Figure S7) and many others. KAAS analysis showed that the assembled transcripts were distributed among several metabolic pathways, which provided the first overview of the Guinea grass transcriptome. Gathering all of the information obtained using the different annotation strategies, we provided initial information about the whole transcriptome of *P. maximum* leaves. Finally, 12,450 unigenes were not annotated in any of the databases compared in this study. Many of these 9,581 sequences were short contigs, of less than 500 bp. We searched for ORFs in this set of non-annotated unigenes and found 243 putative coding regions. Future analysis of this small dataset may reveal potential unknown genes in *P. maximum*.

### Abundance Estimation

**Figure 3:**
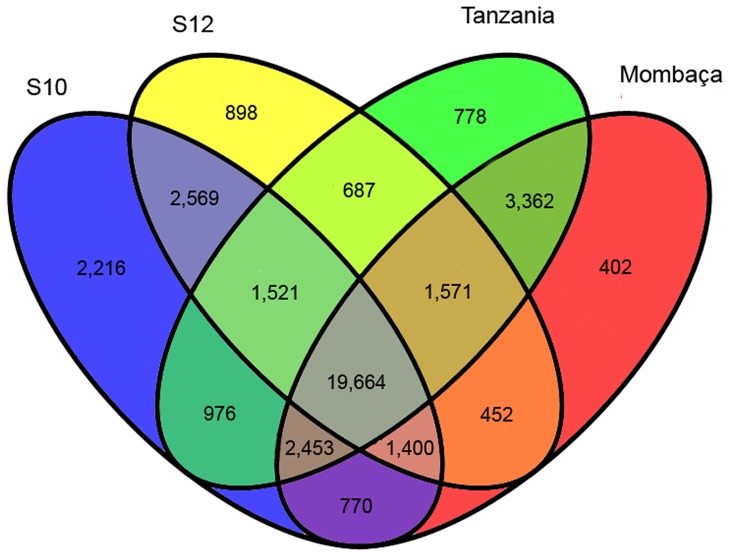
Shared and unique unigenes of *Panicum maximum* determined based on RSEM analysis.

The assembled transcripts were mapped using Bowtie [25], and their respective read abundances (FPKM) were estimated by RSEM [30]. Reads from each genotype were mapped individually and in combination against the reference transcriptome. We used a FPKM cut-off value of 0.5 to define the unigenes expressed in different samples sequenced in this study. Based on these values, we determined which unigenes were unique or shared among the genotypes, as shown in Figure 3. The samples shared a total of 19,664 unigenes, and the S10 genotype displayed a higher number of unique transcripts (2,216). We also selected the ten most abundant transcripts for a brief description (Table 3). The mean FPKM value of the unigenes was approximately 21. The first and third most represented unigenes were ribosomal RNA proteins, members of the translational machinery required for general protein synthesis. Among the more abundant unigenes, four were associated with photosynthesis and carbon fixation in leaves; carbonic anhydrase was the second most represented unigene (10,021 FPKM). Carbonic anhydrase catalyzes the interconversion of CO_2_ and HCO3^−^ and is assumed to play an important role in photosynthesis [44]. Phosphoenolpyruvate carboxylase (PEPC) and pyruvate phosphate dikinase (PPDK) are also key enzymes in the C_4_ photosynthetic pathway. Chlorophyll a-b binding proteins are components of light-harvesting complexes in plants that are crucial for the photosynthesis process [45]. We expected these carbon fixation enzymes to be abundant because the mRNA was extracted from the leaves. Two metallothionein-like proteins were also well represented. Metallothioneins (MTs) are cysteine-rich proteins that coordinate heavy metal atoms [46]. Plant MTs bind to metals with high affinity, and the respective genes are up-regulated in the presence of metal molecules. Because of these characteristics, plant MTs are believed to be involved in cellular metal homeostasis and tolerance mechanisms [47]. Furthermore, two undescribed proteins detected in other grasses were identified among the ten most abundant transcripts among unigenes: a stem-specific protein and a protein from the chloroplastic ycF76 family. Further studies are needed to determine the biological functions of these proteins.

**Table 3:** The 10 most abundant transcripts found in the Guinea grass leaf transcriptome.

Putative gene	E-value	FPKM	UniProtKB
Transcript antisense to ribosomal RNA protein 2	2.00E-016	10702.87	gi|74630365|sp|Q8TGM7.1|ART2_YEAST
Carbonic anhydrase	1.00E-121	10021.35	gi|729003|sp|P40880.1|CAHC_HORVU
Transcript antisense to ribosomal RNA protein 1	1.00E-011	7523.47	gi|74644329|sp|Q8TGM6.1|TAR1_YEAST
Uncharacterized protein ycf76	2.00E-050	7410.46	gi|75121187|sp|Q6ENQ6.1|YCF76_SACOF
Stem-specific protein TSJT1	1.00E-028	5228.23	gi|136452|sp|P24805.1|TSJT1_TOBAC
Phosphoenolpyruvate carboxylase 1	0.0	5043.19	gi|115608|sp|P04711.2|CAPP1_MAIZE
Chlorophyll a-b binding protein	4.00E-139	4389.51	gi|122246902|sp|Q10HD0.1|CB23_ORYSJ
Pyruvate, phosphate dikinase 1	0.0	4164.02	gi|193806357|sp|P11155.2|PPDK1_MAIZE
Metallothionein-like protein 3A	2.00E-008	3959.71	gi|158512839|sp|A2 WLS0.1|MT3A_ORYSI
Metallothionein-like protein 1A	8.00E-009	3775.59	gi|158513336|sp|A2ZH20.1|MT1A_ORYSI

### Genes of Interest

To describe the genes of interest, we used sequences with associated KO terms derived from the KAAS [29] analysis. Most of the enzymes in the C_4_ photosynthetic pathway (Table 4) and in cellulose and lignin production (Table 5) were identified. These pathways are important for efficient beef cattle grazing and the potential for biomass accumulation for energy generation. However, further studies, including specific molecular and proteomic analysis procedures, are required to validate these predictions.

**Table 4:** List of genes comprising the C_4_ photosynthetic pathway found among *P. maximum* unigenes.

Putative gene	KEGG orthology	Enzyme code	Unigenes
Carbonic anhydrase	K01673/K01674	4.2.1.1	8
Phosphoenolpyruvate carboxylase	K01595	4.1.1.31	12
Aspartate aminotransferase, cytosolic	K14454	2.6.1.1	1
Phosphoenolpyruvate carboxykinase	K01610	4.1.1.49	3
Pyruvate kinase	K00873	2.7.1.40	10
Alanine transaminase	K00814	2.6.1.2	1
Malate dehydrogenase (NADP+)	K00051	1.1.1.82	3
Malate dehydrogenase (oxaloacetate-decarboxylating)	K00029	1.1.1.40	4
Pyruvate, orthophosphate dikinase	K01006	2.7.9.1	1
Malate dehydrogenase	K00025	1.1.1.37	1
Malate dehydrogenase (decarboxylating)	K00028	1.1.1.39	2
Ribulose-bisphosphate carboxylase	K01602	4.1.1.39	1
Aspartate aminotransferase, chloroplastic	K00811	2.6.1.1	2

**Table 5:** List of genes comprising the cellulose and lignin pathways found among *P. maximum* unigenes.

Putative gene	KEGG orthology	Enzyme code	Unigenes
**Cellulose biosynthesis**			
UTP–glucose-1-phosphate uridylyltransferase	K00963	2.7.7.9	2
Sucrose synthase	K00695	2.4.1.13	3
Cellulose synthase A	K10999	2.4.1.12	21
Sterol 3beta-glucosyltransferase	K05841	2.4.1.173	4
**Lignin biosynthesis**			
Phenylalanine ammonia-lyase	K10775	4.3.1.24	3
Trans-cinnamate 4-monooxygenase	K00487	1.14.13.11	4
4-Coumarate-CoA ligase	K01904	6.2.1.12	10
Cinnamoyl-CoA reductase	K09753	1.2.1.44	5
Cinnamyl alcohol dehydrogenase	K00083	1.1.1.195	6
Peroxidase	K00430	1.11.1.7	21
Shikimate O-hydroxycinnamoyltransferase	K13065	2.3.1.133	8
Caffeoyl-CoA O-methyltransferase	K00588	2.1.1.104	2
Ferulate-5-hydroxylase	K09755	1.14.-.-	2

#### C_4_ photosynthesis pathway

C_4_ plants such as grasses express high levels of carbonic anhydrase and PEP carboxylase (PEPC) for initial CO_2_ fixation in the cytoplasm [48]. These two enzymes showed high FPKM values (10,021 for CA and 5,043 for PEPC) and were the second and fifth most abundant transcripts, respectively, among the unigenes. The carbonic anhydrases form a family of enzymes that catalyze the interconversion of CO_2_ and H_2_O to bicarbonate (HCO3^−^) and protons. PEPC catalyzes the addition of available bicarbonate to phosphoenolpyruvate (PEP) to form oxaloacetate (OAA) [49]. PEPC has high transcript abundance in *P. virgatum* [39]. OAA is either reduced to malate by NADP-malate dehydrogenase (NADP-MDH) or transaminated to aspartate by aspartate aminotransferase (AspAT). We were only able to find the cytosolic AspAT based on KAAS analysis. A similar distribution was observed in *P. virgatum*; the majority of the transcripts were cytosolic, and only one mitochondrial transcript was identified [39]. The resulting C_4_ acid formed from NADP-MDH or AspAT is then decarboxylated to release CO_2_ in the presence of Rubisco, which is the key enzyme in the C_3_ photosynthetic pathway [49], [50]. The FPKM value for Rubisco was also high (2,530). The decarboxylation reaction is catalyzed by one or more of the following three enzymes: NADP-malic enzyme (NADP-ME), NAD-malic enzyme (NAD-ME) and phosphoenolpyruvate carboxykinase (PEPCK). C_4_ plants are classified into three subtypes depending on their major decarboxylation enzyme [49]. *P. maximum* is considered a PEPCK-type enzyme, and as expected, we found a higher (2,714) FPKM value for this enzyme than for the other two enzymes. The pyruvate generated by decarboxylation is used by pyruvate orthophosphate dikinase (PPDK) to recover the phosphoenolpyruvate (PEP) levels in chloroplasts. PPDK is considered one of the most abundant enzymes in nature [48] and was the eighth most abundant transcript here, with an FPKM value of 4,130.

#### Cellulose biosynthesis

Plant cell walls are complex structures composed of polysaccharides, proteins and lignins [50]. Cell wall constituents are of great importance for livestock grazing and various industrial applications such as biofuel production. The ability to release the carbohydrates from the cell wall for both applications is becoming more important [51]. Among the plant cell wall polysaccharides, cellulose is considered the main component and is a key substrate for livestock foraging and industrial applications [50]. Usually, biofuels are derived from starch-abundant species such as corn or sugarcane. Cellulose-rich plants such as Miscanthus and switchgrass are currently being investigated as alternatives [52]. We searched for homologues of cellulose biosynthesis enzymes using KO terms associated with the unigenes assembled in this work. Cellulose is synthesized by large multimeric cellulose synthase (CesA) complexes. Currently, the only known components of these complexes are the cellulose synthase proteins [50]. We found 21 transcripts with CesA-related KO terms. Among these transcripts, the highest FPKM value was 213. CesA complexes use UDP-glucose (UDP-Glc) as the activated sugar donor for b-1,4 glucan chain polymerization. UDP-Glc can be produced by sucrose synthase (SuSy) or UDP-glucose pyrophosphorylase (UGPase) [50]. We found three representative SuSy and two UGPase transcripts, with FPKM values of 51 and 189, respectively. Because we extracted RNA from the leaves, we expected that SuSy and UGPase would be expressed, although SuSy is mainly expressed in sink tissues [50]. Steryl glycosides may act as initiators of cellulose polymerization [53] and/or alter membrane conditions for cellulose synthesis [54]. Among the unigenes, four sequences were assigned KO terms belonging to sterol beta-glucosyltransferase enzymes. Invertases have been proposed to provide carbon for cellulose production in non-photosynthetic cells [55], and they were not found among the *P. maximum* leaf unigenes described here.

#### Lignin biosynthesis

Lignin is one of the most abundant organic polymers on Earth, exceeded only by cellulose, and it constitutes approximately one-third of the non-fossil organic carbon and one-fourth to one-third of the dry mass of existing wood [56]. Lignin limits cell-wall digestibility by livestock, modulating the energy availability of forage crops in beef and dairy production [57], and it is also a limiting factor in the conversion of plant biomass to pulp or biofuels [51]. A reduction in lignin concentration would have positive effects on both grazing and the energetic use of grasses because the lignin and cellulose/hemicellulose within the lignocellulose constitute major obstacles to these uses [50]. Lignin is mainly synthesized from hydroxycinnamyl alcohols (or monolignols), coniferyl alcohol, sinapyl alcohol and p-coumaryl alcohol [51]. The monolignols, i.e., guaiacyl (G), syringyl (S) and p-hydroxyphenyl (H) units, are then incorporated into the lignin polymer. H-units are slightly more prevalent in grasses [56]. Among the unigenes of Guinea grass, we associated KO terms with all enzymes involved in monolignol formation (Table 5), with the exception of p-coumarate 3-hydroxylase (C3H) and caffeic acid O-methyltransferase (COMT). After the monolignols are transported to the cell wall, lignin is formed through the dehydrogenation of these molecules. The dehydrogenation reaction has been associated with peroxidases and laccases [56]. Among the unigenes, 18 sequences were associated with peroxidase function, but no laccases were found. The abundances of most enzymes in the lignin pathway found in this study were similar (mean of ∼87 FPKM). However, phenylalanine ammonia-lyase (PAL) was more abundant, at 544 FPKM. This enzyme is up-regulated by high levels of phenylalanine in cells of *Pinus taeda* [58].

### Putative Marker Discovery

Transcriptome sequencing provides valuable resources for the development of molecular markers, mostly because of the high quantity of the generated data, in which different types of polymorphisms (e.g., SNPs and SSRs) can be observed. These markers can be tested for potential utility as molecular markers for population genetics, linkage mapping and comparative genomics studies [59]. Here, we investigated two types of putative markers from *P. maximum* leaves: microsatellites and single nucleotide polymorphisms. Both types of markers need future validation for practical use in *P. maximum* breeding and research.

#### SSR discovery

Assembled Guinea grass contigs were analyzed to identify SSRs. The distributions of tandem repeats are shown in Table 6. Among the 38,192 unigenes, MISA [31] found 4,270 sequences (11.1%) containing 5,035 SSRs using a minimum length criterion of 12 bp, or approximately 1 SSR per 6 kb. This SSR value is slightly higher than that in switchgrass (8.8%) [39], similar to that in *Dendrocalamus latiflorus* (12.8%) [38] and higher than those in expressed sequences in other grasses (1.5–4.7%) [60] or plants in general (1–5%) [61]. A survey of bacterial artificial chromosome libraries of *P. virgatum* revealed one SSR per 5.2-kb [43]. The repeat size distribution analysis indicated that trinucleotides represented 86% of the microsatellite motifs found in *P. maximum* unigenes. This SSR value was higher than those of *P. virgatum* (48%–55%) [39], [43], Ma bamboo (56%) [38] and barley (56%) [31] and similar to that of the red algae *Pyropia haitanensis* (87.1%) [62]. The CCG/CGG motif was the most common trinucleotide repeat (28.8% of trinucleotide SSRs). This particular motif is also the most abundant trinucleotide repeat in *P. virgatum* [39], [43], *P. haitanensis* [62] and *H. vulgare* [31]. Regarding the dinucleotide repeats (8.9% of total SSRs), the AG/CT motif represented 54.3% of this class. Tetra-, penta- and hexanucleotide repeats were less abundant (Table 6).

**Table 6:** Summary of putative SSRs found in *P. maximum* unigenes.

Nucleotiderepeat	Differentmotifs	Number ofSSRs	% of totalSSRs
Dinucleotide	4	451	8.9%
Trinucleotide	10	4333	86%
Tetranucleotide	30	184	3.6%
Pentanucleotide	29	44	0.8%
Hexanucleotide	23	23	0.4%
Total	96	5035	100%

#### SNP markers

**Figure 4:**
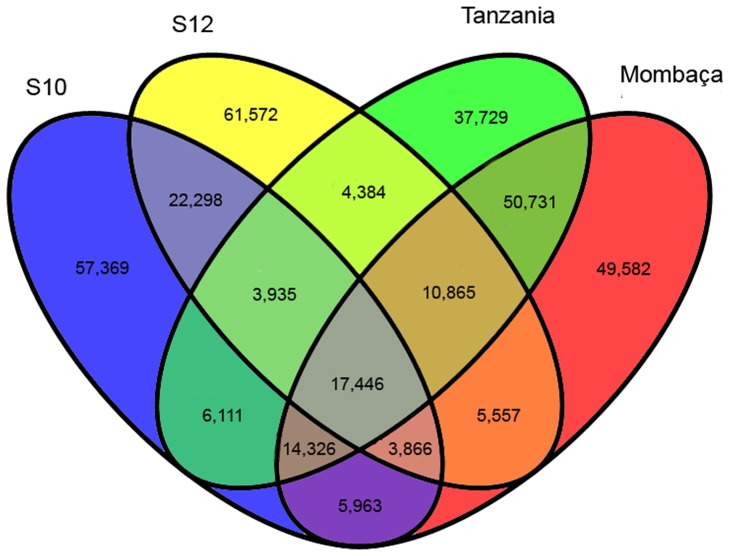
Shared and unique putative SNPs found in *Panicum maximum* unigenes.

For SNP calling, BWA [33] was used to map the reads of each sample to the reference transcriptome. Freebayes [35] detected a total of 346,456 putative SNP positions in the unigenes using previously detailed parameters, as shown in Table 7. This value corresponds to approximately one SNP per 90 bp of unique transcript sequence (1/87), similar to the ratio in rice [63]. Transitions were approximately 1.93 times more abundant than transversions (Table 7). Transitions are generated by oxidative deamination and tautomerization. Although there are twice as many possible transversions than transitions, transitions are usually more common in genomes, possibly because of the molecular mechanisms from which they originate [64]. Similarly, transitions are more easily tolerated in natural selection because they are more likely to generate synonymous mutations in coding sequences than are transversions [65]. Among the detected variants, 231,589 (69.09%) were in predicted open reading frames. Based on the RSEM distinction of shared and unique transcripts among the four *P. maximum* genotypes, we determined the intersection of the putative SNPs found using VCFtools [36], which is shown in Figure 4. Considering the unique SNP positions, we found 49,582 variants for Mombaça, 57,369 for S10, 61,572 for S12 and 37,729 for Tanzania. These unique putative markers will be important for Guinea grass breeding, as the sampled genotypes are distinct for several economically important agronomic characteristics. We identified 17,446 SNPs that were present in all genotypes (Figure 4). Additionally, we determined the numbers of SNPs in the key pathways described in this work. The C_4_ pathway contained 1,159 SNPs, whereas cellulose biosynthesis accounted for 491 SNPs, and lignin biosynthesis accounted for 924 SNPs. Prior to this study, there were no SNPs available for *P. maximum*; these markers may thus represent a useful new tool for marker-assisted selection in Guinea grass breeding.

**Table 7:** Summary of putative SNPs found in *P. maximum* unigenes.

SNP type	Count
**Transitions**	228,468
A-G/G-A	113,075
C-T/T-C	115,393
**Transversions**	117,988
A-C/C-A	29,403
A-T/T-A	26,310
T-G/G-T	29,035
G-C/C-G	33,240
Total	346,456

## Supporting Information

Figure S1 **Top-hit species distribution among NCBI nr BLASTx hits.**
(TIF)

Figure S2 **Functional classification of** ***Panicum maximum*** **unigenes based on COG classes.**
(TIF)

Figure S3 **General COG and GO classification of** ***Panicum maximum*** **unigenes.** (A) General COG classification (B) General GO classification.
(TIF)

Figure S4 **Glycolysis and gluconeogenesis KEGG pathway.** Unigenes with associated KEGG orthology terms are shown in red.
(TIF)

Figure S5 **Photosynthesis KEGG pathway.** Unigenes with associated KEGG orthology terms are shown in red.
(TIF)

Figure S6 **Carbon fixation KEGG pathway.** Unigenes with associated KEGG orthology terms are shown in red.
(TIF)

Figure S7 **Phenylpropanoid biosynthesis KEGG pathway.** Unigenes with associated KEGG orthology terms are shown in red.
(TIF)

Table S1 **Statistics of sequenced data for each** ***P. maximum*** **genotype.**
(DOC)
