# Supplementary figures and images for: De Novo Transcriptome Assembly for the Tropical Grass Panicum maximum Jacq
Source: PLoS One. 2013 Jul 29;8(7):e70781. doi: 10.1371/journal.pone.0070781 (PMC3726610; doi:10.1371/journal.pone.0070781)

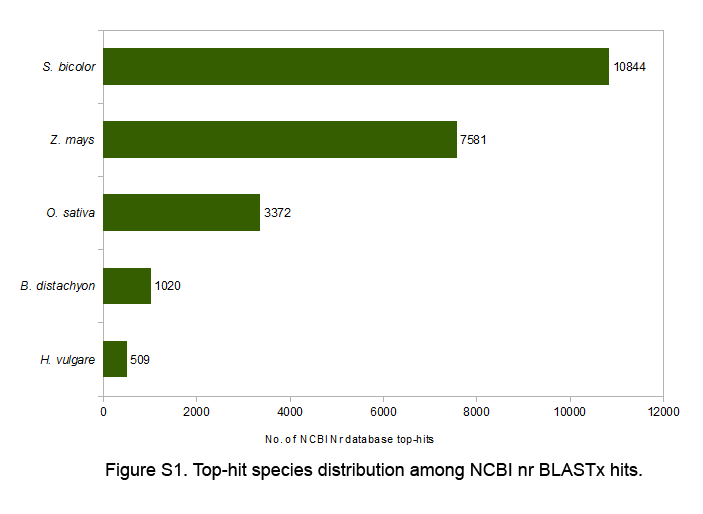

Supplement: Figure S1 — Top-hit species distribution among NCBI nr BLASTx hits. (TIF) [file pone.0070781.s001.tif]

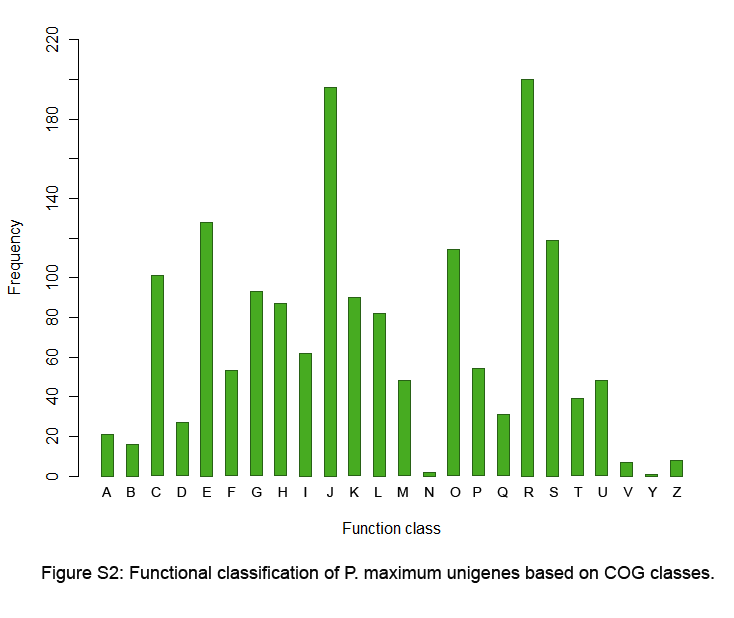

Supplement: Figure S2 — Functional classification of Panicum maximum unigenes based on COG classes. (TIF) [file pone.0070781.s002.tif]

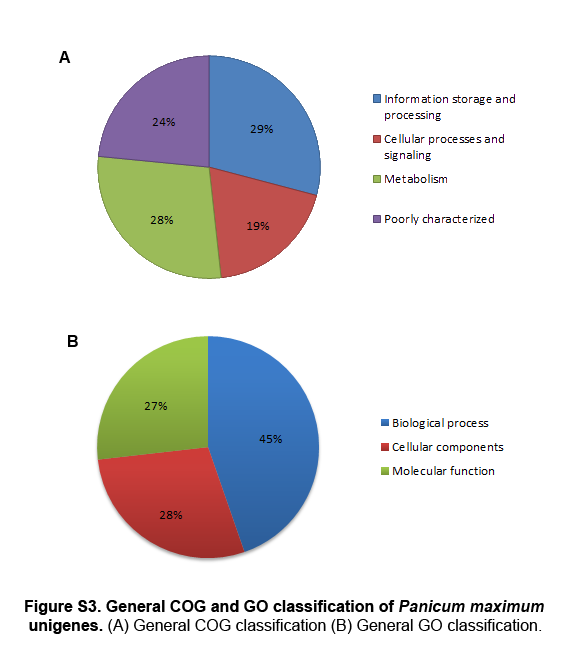

Supplement: Figure S3 — General COG and GO classification of Panicum maximum unigenes. (A) General COG classification (B) General GO classification. (TIF) [file pone.0070781.s003.tif]

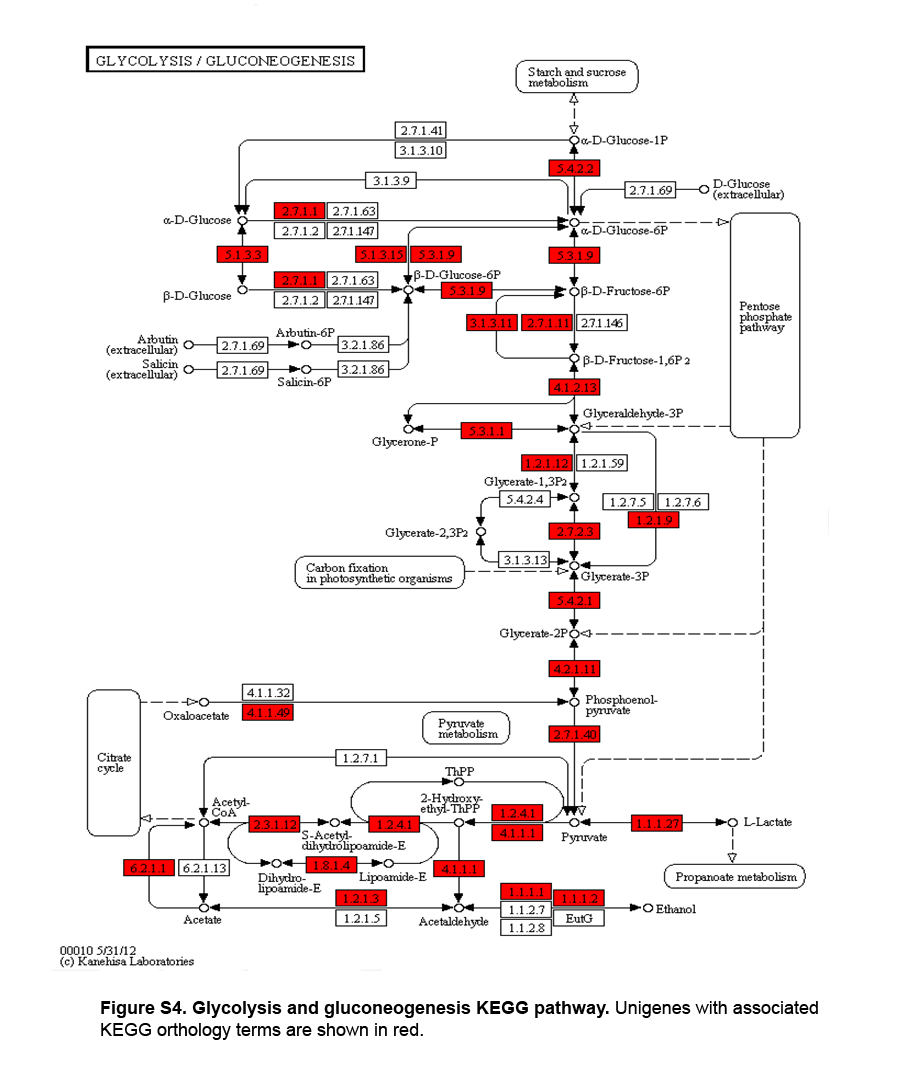

Supplement: Figure S4 — Glycolysis and gluconeogenesis KEGG pathway. Unigenes with associated KEGG orthology terms are shown in red. (TIF) [file pone.0070781.s004.tif]

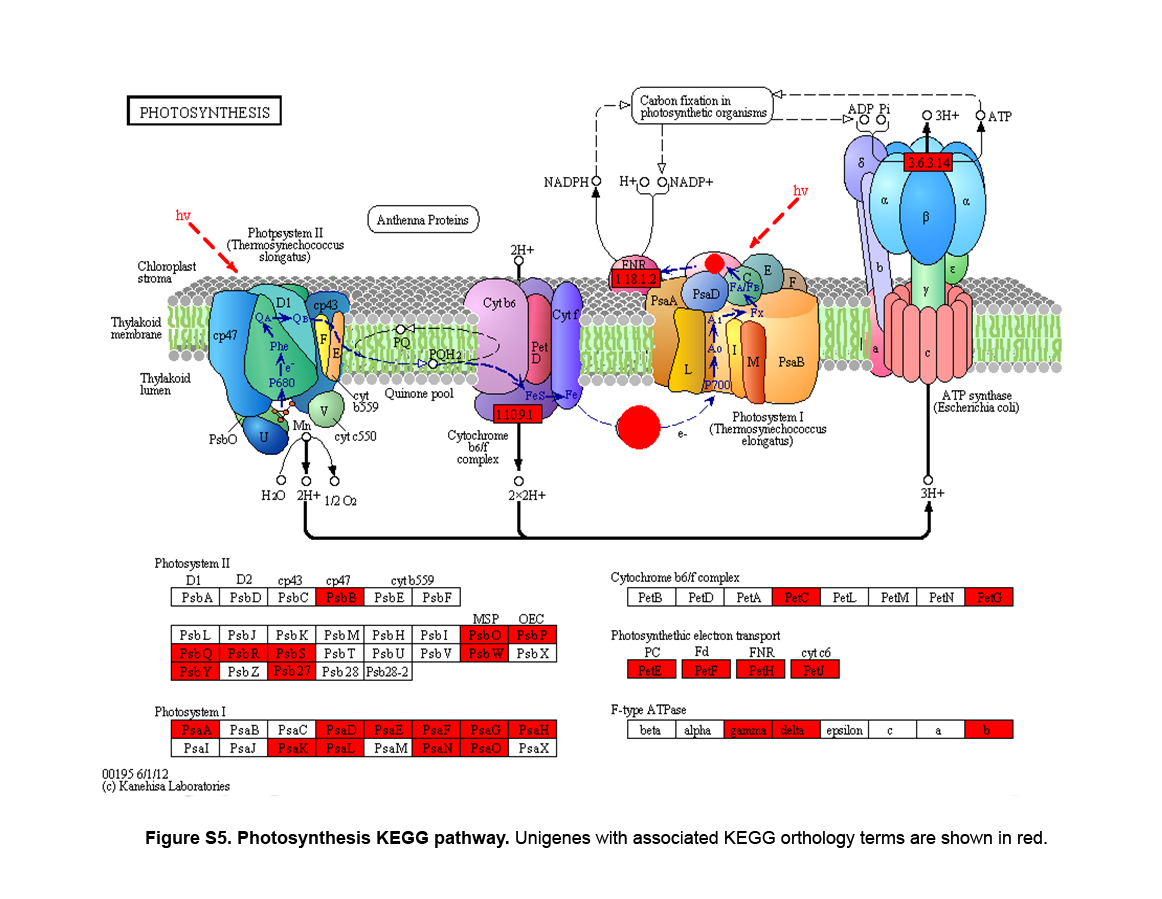

Supplement: Figure S5 — Photosynthesis KEGG pathway. Unigenes with associated KEGG orthology terms are shown in red. (TIF) [file pone.0070781.s005.tif]

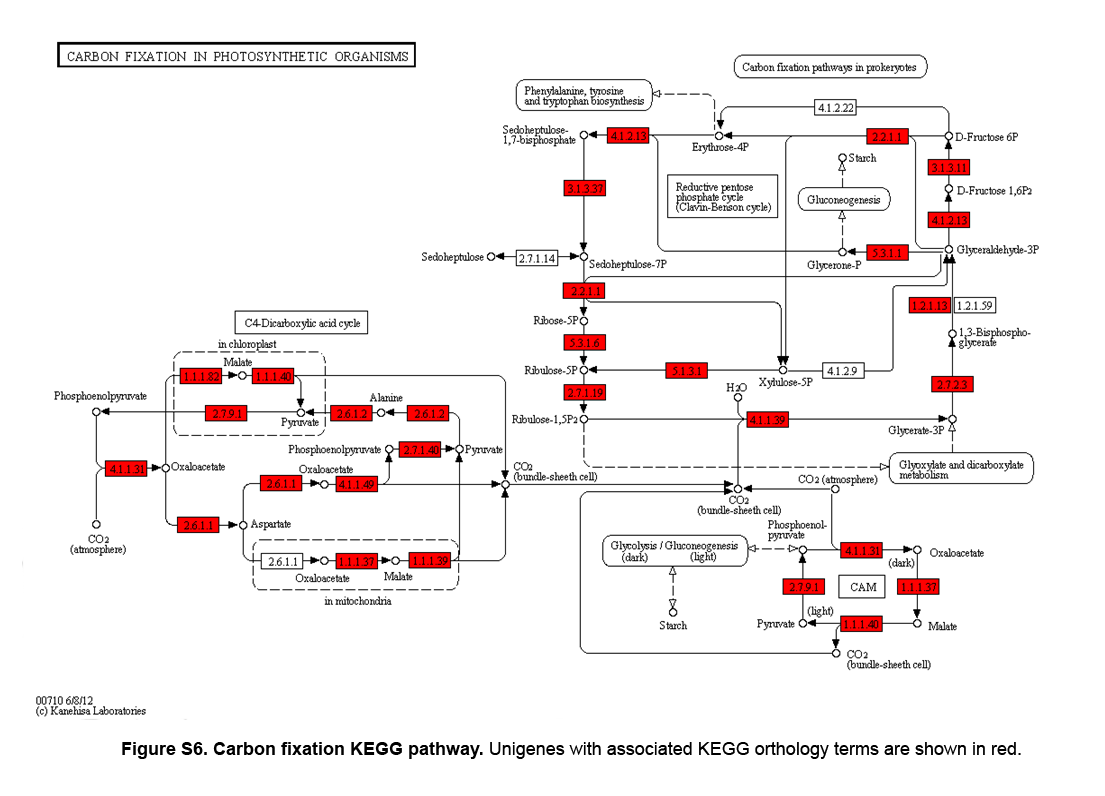

Supplement: Figure S6 — Carbon fixation KEGG pathway. Unigenes with associated KEGG orthology terms are shown in red. (TIF) [file pone.0070781.s006.tif]

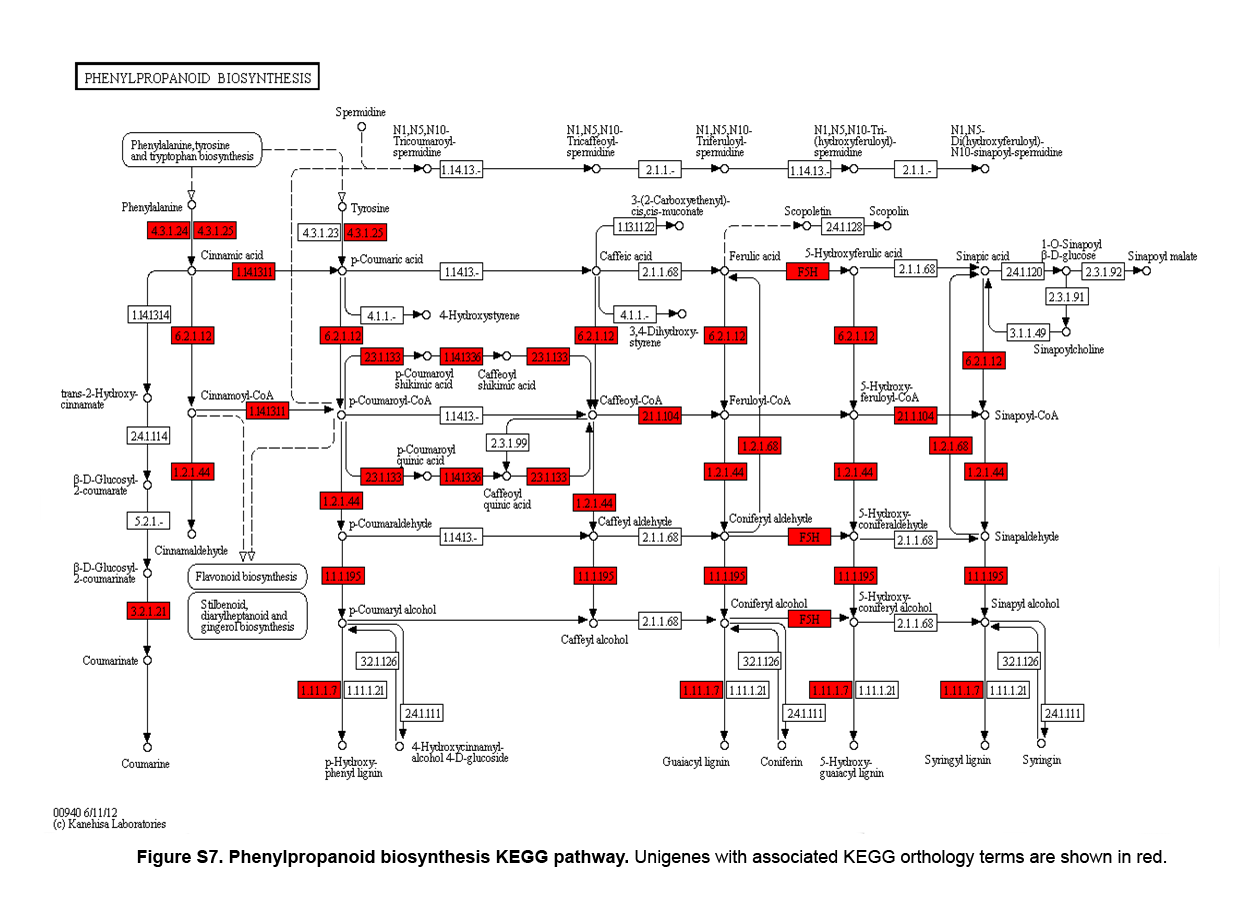

Supplement: Figure S7 — Phenylpropanoid biosynthesis KEGG pathway. Unigenes with associated KEGG orthology terms are shown in red. (TIF) [file pone.0070781.s007.tif]
