# Supplementary material for: De Novo Transcriptome Assembly for the Tropical Grass Panicum maximum Jacq
Source: PLoS One. 2013 Jul 29;8(7):e70781. doi: 10.1371/journal.pone.0070781 (PMC3726610; doi:10.1371/journal.pone.0070781)
Supplement: Table S1 — Statistics of sequenced data for each P. maximum genotype. (DOC) [file pone.0070781.s008.doc]

**Table S1: Statistics of sequenced data for each *P. maximum* genotype.**

|  | **S10** | **S12** | **Mombaça** | **Tanzania** |
| --- | --- | --- | --- | --- |
| Total raw reads | 41,459,861 | 37,275,090 | 47,523,787 | 41,794,980 |
| Total data | 3.31 Gb | 2.98 Gb | 3.80 Gb | 3.34 Gb |
| Total high-quality reads | 30,799,030 | 27,633,430 | 33,178,507 | 29,227,369 |
| High-quality reads % | 74.29% | 74.13% | 69.81% | 69.93% |
